# A Novel Class of Tyrosyl-DNA Phosphodiesterase 1 Inhibitors That Contains the Octahydro-2*H*-chromen-4-ol Scaffold

**DOI:** 10.3390/molecules23102468

**Published:** 2018-09-26

**Authors:** Nikolai S. Li-Zhulanov, Alexandra L. Zakharenko, Arina A. Chepanova, Jinal Patel, Ayesha Zafar, Konstantin P. Volcho, Nariman F. Salakhutdinov, Jóhannes Reynisson, Ivanhoe K. H. Leung, Olga I. Lavrik

**Affiliations:** 1 N. N. Vorozhtsov Novosibirsk Institute of Organic Chemistry, Siberian Branch of the Russian Academy of Sciences, 9, Akademika Lavrentieva Ave., Novosibirsk 630090, Russia; lizhulanov@mail.ru (N.S.L.-Z.); anvar@nioch.nsc.ru (N.F.S.); 2 Department of Natural Sciences and Institute of Medicine and Psychology, Novosibirsk State University, Pirogova 2, Novosibirsk 630090, Russia; 3 Novosibirsk Institute of Chemical Biology and Fundamental Medicine, Siberian Branch of the Russian Academy of Sciences, 8, Akademika Lavrentieva Ave., Novosibirsk 630090, Russia; sashaz@niboch.nsc.ru (A.L.Z.); arinachepanova@mail.ru (A.A.C.); lavrik@niboch.nsc.ru (O.I.L.); 4 School of Chemical Sciences, The University of Auckland, Private Bag 92019, Victoria Street West, Auckland 1142, New Zealand; jpat649@aucklanduni.ac.nz (J.P.); azaf173@aucklanduni.ac.nz (A.Z.)

**Keywords:** anticancer agent, Tdp1 inhibitor, DNA repair enzyme, synthesis, biochemical assay, molecular modeling, chemical space, structural-activity relationships

## Abstract

Tyrosyl-DNA phosphodiesterase 1 (Tdp1) is a DNA repair enzyme that mends topoisomerase 1-mediated DNA damage. Tdp1 is a current inhibition target for the development of improved anticancer treatments, as its inhibition may enhance the therapeutic effect of topoisomerase 1 poisons. Here, we report a study on the development of a novel class of Tdp1 inhibitors that is based on the octahydro-2*H*-chromene scaffold. Inhibition and binding assays revealed that these compounds are potent inhibitors of Tdp1, with IC_50_ and *K*_D_ values in the low micromolar concentration range. Molecular modelling predicted plausible conformations of the active ligands, blocking access to the enzymatic machinery of Tdp1. Our results thus help establish a structural-activity relationship for octahydro-2*H*-chromene-based Tdp1 inhibitors, which will be useful for future Tdp1 inhibitor development work.

## 1. Introduction

Tyrosyl-DNA phosphodiesterase 1 (Tdp1) is an enzyme that belongs to the phospholipase D superfamily [1,2]. Tdp1 functions as a DNA repair enzyme in cells, in which it removes stalled topoisomerase 1 (Top1)–DNA complexes by catalysing the hydrolysis of phosphodiester bonds between the tyrosine residue of Top1 and the 3′-phosphate of DNA [3]. Top1 is a current inhibition target for chemotherapeutic agents [4]. However, Tdp1 also repairs DNA damage that is caused by Top1 inhibitors. Thus, the inhibition of Tdp1 may enhance the efficacy of Top1 poisons, potentially reducing the side effects of chemotherapeutic treatments and enabling a lower dosage to be used.

**Figure 1:**
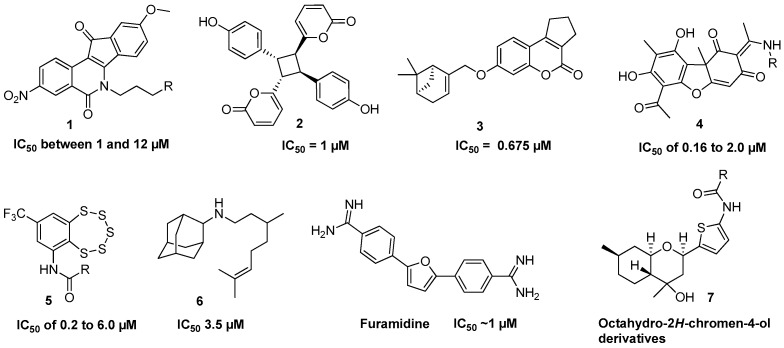
Structures of reported Tdp1 inhibitors **1**–**6**, commercial Tdp1 inhibitor Furamidine, and octahydro-2*H*-chromen-4-ol derivatives **7** that we investigated in this study.

Given the potential of Tdp1 inhibition in enhancing the efficacy of anticancer treatments, several types of Tdp1 inhibitors have been developed [5,6,7,8,9,10,11,12,13,14]. Most of these inhibitors show a good to moderate inhibition potency, with IC_50_ values in the concentration range between 0.1 and 20 μM. These include 7-azaindenoisoquinoline derivatives **1** such as triple Top1/Tdp1/Tdp2 inhibitors [5]; achyrodimer F **2**, which is a dimeric cyclobutane metabolite from an Australian fungus of the *Cortinariaceae* family [6]; 7-hydroxycoumarins derivatives **3** that contain aromatic or monoterpene substituents [7]; usnic acid derivatives, such as **4** with an enamine moiety [8]; compounds that contain the benzopentathiepine moiety **5** [9]; aminoadamantane-containing monoterpene-derived compound **6** [10]; and furamidine (NSC 305831), which is a commercial Tdp1 inhibitor [11] (Figure 1), as well as the other less potent inhibitors of various chemical classes [12,13,14].

Octahydro-2*H*-chromene is an oxygen-containing heterocycle with a tetrahydropyran moiety. This structural scaffold occurs in many important natural products and biologically-active molecules [15,16]. For example, compounds that contain octahydro-2*H*-chromen-4-ol were found to exhibit antiviral and analgesic activities [17,18]. The synthesis of molecules that contain the octahydro-2*H*-chromene scaffold has been investigated extensively. In particular, Prins cyclisation was found to be a flexible method for the synthesis of octahydro-2*H*-chromenes derivatives. Typically, the reaction involves a homoallylic alcohol and a carbonyl compound being catalysed by Brønsted or Lewis acids. The most commonly used catalysts in this transformation are montmorillonite clays or Lewis acids such as Sc(OTf)_3_, BF_3_*Et_2_O, or I_2_ [19,20,21,22]. Montmorillonite clays were also found to be effective catalysts for the synthesis of octahydro-2*H*-chromenes with heterocyclic motifs [23,24,25,26]. Although aldehydes containing amino groups cannot be involved in such reactions because of catalyst poisoning [26], in principle, such products can be obtained through reactions with nitro-aromatic aldehydes, followed by the reduction of the nitro group. For example, we have successfully obtained nitro-containing octahydro-2*H*-chromene from 5-nitro-thiophene-2-carbaldehyde in our previous work [18]. Moreover, the electron-rich thiophene ring may facilitate the reduction of the nitro group into an amino group, which is especially important for compounds that are relatively labile, such as those that contain a tert-hydroxy group. Thus, we reasoned that, due to the versatility and ease of synthesis, thiophene-2-carbaldehyde is an ideal starting point for the synthesis and derivatisation of compounds that contain the octahydro-2*H*-chromene scaffold.

By using a range of octahydro-2*H*-chromen-4-ol derivatives with different amide motifs **7** (Figure 1), preliminary molecular modelling studies were performed. Our modelling results suggested that these derivatives may bind to Tdp1. We therefore reasoned that octahydro-2*H*-chromen-4-ol may be a useful scaffold for the development of new Tdp1 inhibitors. Herein, we report our work on the synthesis of a structural series of octahydro-2*H*-chromen-4-ol derivatives as potential Tdp1 inhibitors. Inhibition and binding assays were performed to evaluate their inhibition potency and binding affinity to Tdp1, respectively. Finally, molecular modelling was performed to predict the plausible binding conformations of these compounds.

## 2. Results and Discussion

### 2.1. Synthesis of Octahydro-2H-chromen-4-ol Derivatives

**Scheme 1:**
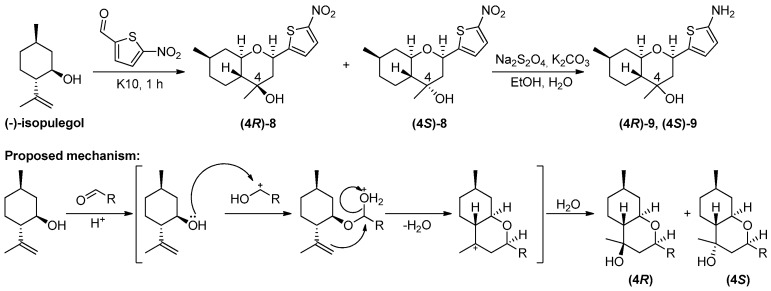
Synthesis of (**4*R***)-**9** and (**4*S***)-**9** compounds and proposed mechanism for the synthesis of **4*R*** and **4*S***, as proposed by Nazimova et al*.* [18].

Nitro compounds **8** with an octahydro-2*H*-chromen-4-ol scaffold were synthesised as a mixture of diastereomers on the C-4 position in a reaction of (-)-isopulegol with 5-nitrothiophene-2-carbaldehyde in the presence of montmorillonite K10 clay (Scheme 1).

The possible mechanism of the transformation depicted in Scheme 1 has been previously proposed [18]. After purification using column chromatography, the individual products (**4*R***)-**8** and (**4*S***)-**8** were obtained with yields of 25% and 28%, respectively. In order to avoid dehydration of the compounds, their reduction was carried out in a slightly alkaline aqueous alcohol solution with sodium dithionite. This resulted in the production of primary amines (**4*R***)-**9** and (**4*S***)-**9** with yields of 60% and 80%, respectively.

**Scheme 2:**
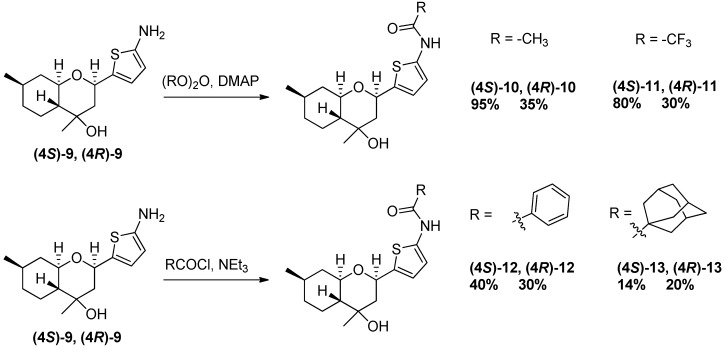
Synthesis of **10–13** amide derivatives of octahydro-2*H*-chromen-4-ol.

The primary amines obtained were selectively acylated with anhydrides and acid chlorides (Scheme 2). Acylation of amine groups solely occurred, even when the acylating reagent was in excess. Using acid chlorides resulted in products of smaller yields compared to acid anhydrides. The desired amide derivatives of octahydro-2*H*-chromen-4-ol **10–13** were purified using column chromatography. In each case, except for the reaction with 1-adamantanecarbonyl chloride, the yield of (**4*S***)-diastereomer was higher than the one of (**4*R***). We supposed that the isolation of (**4*R***)-diastereomers was less successful, likely due to their instability during column chromatography.

The structure of the compounds was determined by nuclear magnetic resonance (NMR) spectroscopy, including ^1^H, ^13^C, ^1^H–^1^H 2D homonuclear correlation spectroscopy (COSY), ^1^H–^13^C 2D heteronuclear single quantum coherence (HSQC), and ^1^H–^13^C 2D heteronuclear multiple bond correlation (HMBC) experiments. As the hydroxyl group of (**4*R***)-isomers **8**–**13** is in the axial position, this causes the ^1^H resonances of neighbouring protons, H-1 and H-3, to shift upfield by ~0.3 ppm and ~0.4 ppm, respectively, when compared with (**4*S***)-isomers. This phenomenon is likely caused by the 1,3-diaxial interaction, which was previously observed in our studies of similar compounds [17,18].

### 2.2. Tdp1 Inhibition and Binding Assays

The inhibition potency of our synthesised compounds against Tdp1 was evaluated by using a real-time oligonucleotide biosensor-based assay [27]. In this assay, Tdp1 catalyses the removal of fluorophore quenchers from the 3′-end of a modified DNA substrate [27]. Fluorescence intensity hence reflects the catalytic activity of the enzyme. Hexadecameric oligonucleotide was selected as a Tdp1 substrate, as it contains 5(6)-carboxyfluorescein (FAM) at the 5′-end and fluorophore quencher BHQ1 (Black Hole Quencher-1) at the 3′-end [9]. This approach was used to determine the inhibitory properties of all the chromenes.

All our synthesised compounds except derivatives **8** and **9** were found to exhibit inhibitory activity against Tdp1 in the low micromolar range (Table 1). Compounds with IC_50_ > 15 μM were considered inactive. The (**4*S***)-diastereomer with the most bulky substituent was found to be the most potent (**13**), with an IC_50_ value of 1.24 μM. In general, the IC_50_ values for the (**4*S***) series are in the IC_50_ of 1.24 to 5.8 µM range. For (**4*R***)-diastereomers, their IC_50_ values were found to be similar.

In addition to the inhibition assay, the binding of our compounds to Tdp1 was also evaluated. An intrinsic protein fluorescence-based assay, which we have previously applied to study the binding of esters combining monoterpenoid and adamantane fragments, was applied [28]. In agreement with the inhibition data, all our compounds were relatively strong binders to Tdp1, with dissociation constants (*K*_D_) in the low μM region (Table 1 and Supplementary Figures S1–S7). Interestingly, all our compounds appeared to bind Tdp1 to more than one binding pocket (i.e., specific and non-specific binding). This is possibly due to the large hydrophobic surface of Tdp1. The slight discrepancy between the measured *K*_D_ and IC_50_ values is likely due to these non-specific binding events.

### 2.3. Molecular Modelling

Both isomers (**4*R***, **4*S***) of the six ligands were docked into Tdp1 (PDB ID: 1MU7) [2,29] using GOLD and all the ligands fitted into the pocket in a similar way with a reasonable predicted affinity for the scoring functions used, as can be seen in Table 2. GoldScore (GS) and, in particular, Chem Piecewise Linear Potential (ChemPLP), scoring functions give higher scores for **10–13** (**4*R***, **4*S***) compared to (**4*R***, **4*S***)-**8** and (**4*R***, **4*S***)-**9**, which is consistent with their IC_50_ values. A similar trend is also observed for ChemScore (CS) and Astex Statistical Potential (ASP), albeit not as pronounced as for GoldScore and ChemPLP. The correlation between the measured affinities and the predicted binding values is reassuring, particularly since ChemPLP is considered to be the most robust function used [30] and strengthens the overall applicability of the modelling.

**Figure 2:**
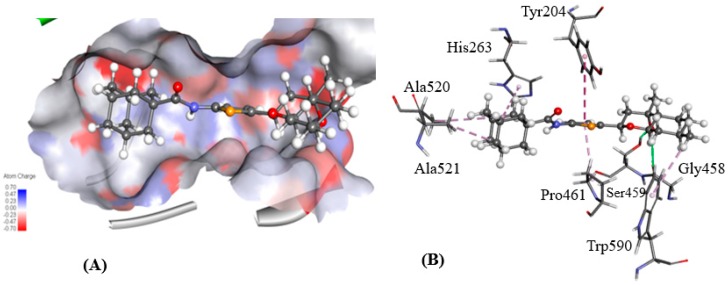
Docked configurations of derivative (**4*S***)-**13** to the substrate binding sites predicted by the ChemPLP algorithm. The protein surface is rendered where partial positive, negative charges, and neutral regions are colored blue, red, and grey, respectively (**A**). The hydrogen bond interactions are depicted as green lines between compound (**4*S***)-**13** and the amino acids residues; Ser459 and Gly458 (**B**). Furthermore, lipophilic contacts (LC) are shown as purple dashed lines with His263, Tyr204, Ala520, Ala521, and Pro461.

The docked configuration of the most active derivative (**4*S***)-**13** is shown in Figure 2. Our docking model revealed that the ligand is in lipophilic contact (LC) with His263, Tyr204, Ala520, Ala521, and Pro461, and exhibited hydrogen bonding with Gly458 and Ser459 via the hydroxyl group. Also, the hydrophobic pocket in the binding site is occupied by the adamantane moiety and the thiophene group occupies a cleft exposed to the water environment, as shown in Figure 2A. Derivative (**4*S***)-**11**, which shows a similar inhibition potency to (**4*S***)-**13**, is predicted to have LC with His263 and Tyr204 in a similar manner to (**4*S***)-**13**. However, (**4*S***)-**11** displayed a hydrogen bonding interaction with His493 through its hydroxyl group, unlike (**4*S***)-**13**. This suggests that the different substituents on the amide group can have an important effect in orienting the ligands in the binding site of the enzyme. It is established that for Tdp1, both His263 and His493 play a key role in its biological function and the binding pocket was defined there [1,7,9]. It can therefore be stated that a plausible binding mode is predicted as the ligands block access to these key amino acid residues.

The calculated molecular descriptors (MW-molecular weight, log P octanol–water partition coefficient, HD–hydrogen bond donors, HA-hydrogen bond acceptors, PSA–polar surface area, and RB–rotatable bonds) for the six isomers (**4*S*, 4*R***) are given in Table S1 (see Supplementary Material). The ligands are relatively average in size, with a molecular weight between 281.4 and 443.6.8 (**4*R*, 4*S***), and nine (**4*R*, 4*S***) derivatives lie in the lead-like space, while others are within the boundaries of the drug-like space (for definition of lead-like, drug-like, and Known Drug Space regions, see ref [31]). The log P values range from 2.3 to 5.1, with only compounds (**4*R*, 4*S***)-**8**, (**4*R*, 4*S***)-**9**, and (**4*R***)-**10** being slightly outside of the ideal drug-like space but within the Known Drug Space. Moreover, the Polar Surface Area (PSA) values also indicate that they lie in the drug-like space.

The SAR and modelling studies clearly indicate that lipophilic R-groups are potentially beneficial for improved binding of this class of compounds to Tdp1. To investigate this possibility, four derivatives with the R-groups α-naphthyl (**14**), β-naphthyl (**15**), isopropyl (**16**), and tert-butyl (**17**) were docked into the binding site and their scores are given in Table 2. Compared with the scores for the most active compounds **11** and **13**, it can be seen from Table 2 that the α-naphthyl substitution gives favourable scores for both GS and ChemPLP, suggesting that this derivative may be a promising derivative to synthesise and test. The other substitutions gave similar or worse scores compared to **11** and **13**. Similar binding poses were predicted for derivatives **14**–**17** as for their **8**–**13** counterparts. Finally, derivatives **14** and **15** have slightly higher Log P values than **13,** but have essentially the same values for the other molecular descriptors (see Table S1 in the SI). 

## 3. Materials and Methods

### 3.1. Chemistry

#### 3.1.1. General Materials and Methods

Chemicals were purchased from commercial sources (Sigma-Aldrich, St. Louis, MO, USA; Acros, Morris Plains, NJ, USA) and used with no further treatment. The K10 clay (Aldrich) was used as the catalyst of the reaction. The clay was dried at 105 °C for 3 h prior to use. Dichloromethane CH_2_Cl_2_ was dried with calcined Al_2_O_3_. For the products structure analysis, GC-MS was used, consisting of an Agilent 7890A gas chromatograph equipped with a quadrupole mass spectrometer Agilent 5975C as a detector. A 30-m quartz column HP-5MS (copolymer 5%–diphenyl–95%–dimethylsiloxane) with an inner diameter of 0.25 mm and the stationary phase film thickness of 0.25 µm was used. Optical rotation parameters: polAAr 3005 spectrometer; CHCl_3_ soln. ^1^H and ^13^C NMR: Bruker DRX-500 apparatus at 500.13 MHz (^1^H) and 125.76 MHz (^13^C), *J* in Hz; structure was determined by analysing the ^1^H NMR spectra, including ^1^H–^1^H double resonance spectra and ^1^H–^1^H 2D homonuclear correlation, J-modulated ^13^C NMR spectra (JMOD), and ^13^C –^1^H 2D heteronuclear correlation with one-bond and long-range spin-spin coupling constants (C–H COSY, ^1^*J*(C,H) = 160 Hz, COLOC, ^2,3^*J*(C,H) = 10 Hz). HR-MS: DFS Thermo Scientific spectrometer in a full scan mode (15-500 m/z, 70 eV electron impact ionisation, direct sample administration).

All product yields are given as pure compounds obtained from the column chromatography. Column chromatography: silica gel (SiO_2_; 60-200 μ; Macherey-Nagel); hexane, solution containing from 0 to 100% EtOAc in hexane, ethanol. The purity of the target compounds was determined by GC-MS methods. The purity of all target compounds reported in this paper exceeds 95%.

**Figure 3:**
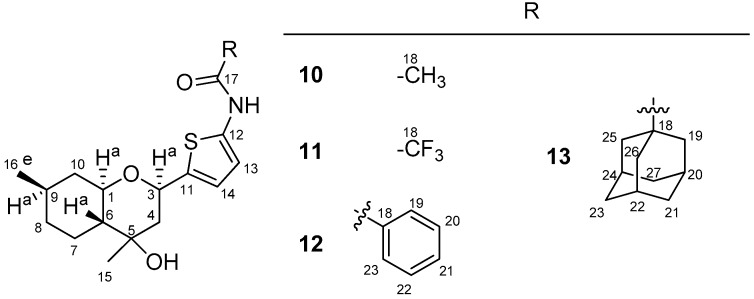
The numeration of the atoms of **8–13** in NMR spectra.

Please note that the numeration of the atoms of products is performed as shown in Figure 3.

#### 3.1.2. Chemical Syntheses

The syntheses and characterisations of *(2R,4aR,7R,8aS)-4,7-Dimethyl-2-(5-nitrothiophen-2-yl)octahydro-2H-chromen-4-ols*, (**4*S***)-**8**, and (**4*R***)-**8**, were reported previously [18].

*(2R,4R,4aR,7R,8aS)-4,7-Dimethyl-2-(5-aminothiophen-2-yl)octahydro-2H-chromen-4-ol*, (**4*R***)-**9**. To the solution of nitro compound (**4*R***)-**8** (0.436 g) in EtOH (20 mL), Na_2_S_2_O_4_ (2.300 g) and K_2_CO_3_ (1.900 g) in water (10 mL) were added, and the mixture was stirred at r.t. for 2 h. After the completion of the reaction, 10 mL of a saturated aq. NaCl solution was added to the reaction mixture and extracted with ethyl acetate (3 × 10 mL). The organic layer was washed with brine (2 × 10 mL) and dried over anhydrous sodium sulphate. The organic layer was concentrated in a rotary evaporator and separated using an SiO_2_ column. Purification resulted in the yield of (**4*R***)-**9** (0.234 g, 60%). ^1^H-NMR (CDCl_3_) (ppm) δ: 0.88–0.97 (m, 1H, Ha-8); 0.92 (d, *J*(16, 9a) = 6.5 Hz, 3H, H-16); 1.05 (ddd, *J*(10a, 10e) = *J*(10a, 9a) = 12.1 Hz, *J*(10a, 1a) = 11.2 Hz, 1H, Ha-10); 1.09–1.20 (m, 2H, Ha-6, Ha-7); 1.24 (s, 3H, H-15); 1.41–1.52 (m, 1H, Ha-9); 1.67 (dd, *J*(4a, 4e) = 13.4 Hz, *J*(4a, 3a) = 11.6 Hz, 1H, Ha-4); 1.72 (dm, *J*(8e, 8a) = 13.1 Hz, other *J* < 3.5 Hz, 1H, He-8); 1.77–1.83 (m, 1H, He-7); 1.95 (dd, *J*(4e, 4a) = 13.4 Hz, *J*(4e, 3a) = 2.4 Hz, 1H, He-4); 1.97 (dm, *J*(10e, 10a) = 12.1 Hz, other *J* < 4.5 Hz, 1H, He-10); 3.21 (ddd, *J*(1a, 10a) = 11.2 Hz, *J*(1a, 6a) = 9.7 Hz, *J*(1a,10e) = 4.2 Hz, 1H, Ha-1); 4.49 (ddd, *J*(3a, 4a) = 11.6 Hz, *J*(3a, 4e) = 2.4 Hz, *J*(3a, 14) = 1.0 Hz, 1H, Ha-3); 5.99 (d, *J*(14, 13) = 3.5 Hz, 1H, H-14); 6.53 (d, *J*(13, 14) = 3.5 Hz, 1H, H-13). ^13^C-NMR (CDCl_3_) (ppm) δ: 72.13 (d, C-1); 70.63 (d, C-3); 47.25 (t, C-4); 69.99 (s, C-5); 49.22 (d, C-6); 22.28 (t, C-7); 34.14 (t, C-8); 31.08 (d, C-9); 40.83 (t, C-10); 150.56 (s, C-11); 132.81 (s, C-12); 122.36 (d, C-13); 107.64 (d, C-14); 27.92 (q, C-15); 22.01 (q, C-16). HR MS: 281.1449 (M^+^, C_15_H_23_O_2_NS^+^; calc. 281.1444). ${\left[\alpha \right]}_{D}^{26.0}$ = +7.2 (c 0.334, EtOH).

*(2R,4S,4aR,7R,8aS)-4,7-dimethyl-2-(5-aminothiophen-2-yl)octahydro-2H-chromen-4-ol*, (**4*S***)-**9**. To the solution of nitro compound (**4*S***)-**8** (0.223 g) in EtOH (20 mL), Na_2_S_2_O_4_ (1.300 g) and K_2_CO_3_ (1.000 g) solution in water (10 mL) was added, and the mixture was stirred at r.t. for 2 h. After the completion of the reaction, 10 mL of a saturated aq. NaCl solution was added to the reaction mixture and extracted with ethyl acetate (3 × 10 mL). The organic layer was washed with brine (2 × 10 mL) and dried over anhydrous sodium sulphate. The organic layer was concentrated in a rotary evaporator and separated on an SiO_2_ column. Purification resulted in the yield of (**4*S***)-**9** (0.167 g, 80%). ^1^H-NMR (CDCl_3_) (ppm) δ: 0.88–0.97 (m, 1H, Ha-8); 0.93 (d, *J*(16, 9a) = 6.5 Hz, 3H, H-16); 1.04 (ddd, *J*(10a, 10e) = *J*(10a, 9a) = 12.1 Hz, *J*(10a, 1a) = 11.2 Hz, 1H, Ha-10); 1.10–1.21 (m, 2H, Ha-6, Ha-7); 1.24 (s, 3H, H-15); 1.41–1.53 (m, 1H, Ha-9); 1.67 (dd, *J*(4a, 4e) = 13.4 Hz, *J*(4a, 3a) = 11.6 Hz, 1H, Ha-4); 1.72 (dm, *J*(8e, 8a) = 13.1 Hz, other *J* < 3.5 Hz, 1H, He-8); 1.77–1.83 (m, 1H, He-7); 1.94 (dd, *J*(4e, 4a) = 13.4 Hz, *J*(4e, 3a) = 2.4 Hz, 1H, He-4); 1.97 (dm, *J*(10e, 10a) = 12.1 Hz, other *J* < 4.5 Hz, 1H, He-10); 3.52 (ddd, *J*(1a, 10a) = 11.2 Hz, *J*(1a, 6a) = 9.7 Hz, *J*(1a,10e) = 4.2 Hz, 1H, Ha-1); 4.82 (ddd, *J*(3a, 4a) = 11.6 Hz, *J*(3a, 4e) = 2.4 Hz, *J*(3a, 14) = 1.0 Hz, 1H, Ha-3); 5.98 (d, *J*(14, 13) = 3.6 Hz, 1H, H-14); 6.51 (d, *J*(13, 14) = 3.6 Hz, 1H, H-13). ^13^C-NMR (CDCl_3_) δ: 76.89 (d, C-1); 72.73 (d, C-3); 47.54 (t, C-4); 69.98 (s, C-5); 49.12 (d, C-6); 22.28 (t, C-7); 34.14 (t, C-8); 31.08 (d, C-9); 40.83 (t, C-10); 150.22 (s, C-11); 131.81 (s, C-12); 120.26 (d, C-13); 108.60 (d, C-14); 27.91 (q, C-15); 22.00 (q, C-16). HR MS: 281.1449 (M^+^, C_15_H_23_O_2_NS^+^; calc. 281.1444). ${\left[\alpha \right]}_{D}^{29.1}$ = −6.25 (c 0.16, EtOH).

*General Procedure for the Preparation of Compounds***10**–**11***.* To the solution of amine **9** (1.0 mmol) in EtOAc, acid anhydride solution (1.0 mmol) in the presence of 4-DMAP (0.01 mmol) was added dropwise, and the mixture was stirred at r.t. for 2 h. After the completion of the reaction, 10 mL of a saturated aq. NaHCO_3_ solution was added to the reaction mixture and extracted with ethyl acetate (3 × 10 mL). The organic layer was washed with brine (2 × 10 mL) and dried over anhydrous sodium sulphate. The organic layer was concentrated in a rotary evaporator and separated using an SiO_2_ column.

*N-(5-((2R,4R,4aR,7R,8aR)-4-Hydroxy-4,7-dimethyloctahydro-2H-chromen-2-yl)thiophen-2-yl)acetamide*, (**4*R***)-**10**. The reaction of amine (**4*R***)-**9** (0.100 g) and acetic anhydride (0.037 g) in the presence of 4-DMAP (0.001 g) for 2 h resulted in the yield of (**4*R***)-**10** (0.031 g, 35%). ^1^H-NMR (CDCl_3_) (ppm) δ: 0.80–0.88 (m, 1H, Ha-8); 0.82 (d, *J*(16, 9a) = 6.5 Hz, 3H, H-16); 0.94 (ddd, *J*(10a, 10e) = *J*(10a, 9a) = 12.1 Hz, *J*(10a, 1a) = 11.2 Hz, 1H, Ha-10); 1.03–1.12 (m, 2H, Ha-6, Ha-7); 1.14 (s, 3H, H-15); 1.33–1.43 (m, 1H, Ha-9); 1.63 (dd, *J*(4a, 4e) = 13.4 Hz, *J*(4a, 3a) = 11.6 Hz, 1H, Ha-4); 1.70–1.89 (m, 4H, He-7, He-8, He-4, He-10); 2.03 (s, 3H, H-18); 3.18 (ddd, *J*(1a, 10a) = 11.2 Hz, *J*(1a, 6a) = 9.7 Hz, *J*(1a, 10e) = 4.2 Hz, 1H, Ha-1); 4.51 (ddd, *J*(3a, 4a) = 11.6 Hz, *J*(3a, 4e) = 2.4 Hz, *J*(3a, 14) = 1.0 Hz, 1H, Ha-3); 6.39 (d, *J*(14, 13) = 3.8 Hz, 1H, H-14); 6.62 (d, *J*(13, 14) = 3.8 Hz, 1H, H-13). ^13^C-NMR (CDCl_3_) (ppm) δ: 77.51 (d, C-1); 72.51 (d, C-3); 49.32 (t, C-4); 70.26 (s, C-5); 51.50 (d, C-6); 22.83 (t, C-7); 34.16 (t, C-8); 31.28 (d, C-9); 41.09 (t, C-10); 136.11 (s, C-11); 138.77 (s, C-12); 110.66 (d, C-13); 120.60 (d, C-14); 22.37 (q, C-15); 20.60 (q, C-16); 167.39 (s, C-17); 21.95 (q, C-18). HR MS: 323.1547 (M^+^, C_17_H_25_NO_3_S^+^; calc. 323.1550). ${\left[\alpha \right]}_{D}^{28.0}$ = +7.48 (c 0.107, EtOH).

*N-(5-((2R,4S,4aR,7R,8aR)-4-Hydroxy-4,7-dimethyloctahydro-2H-chromen-2-yl)thiophen-2-yl)acetamide*, (**4*S***)-**10**. The reaction of amine (**4S**)-**9** (0.040 g) and acetic anhydride (0.015 g) in the presence of 4-DMAP (0.001 g) for 2 h resulted in the yield of (**4S**)-**10** (0.043 g, 95%). ^1^H-NMR (CDCl_3_) (ppm) δ: 0.80–0.88 (m, 1H, Ha-8); 0.82 (d, *J*(16, 9a) = 6.5 Hz, 3H, H-16); 0.94 (ddd, *J*(10a, 10e) = *J*(10a, 9a) = 12.1 Hz, *J*(10a, 1a) = 11.2 Hz, 1H, Ha-10); 1.03–1.12 (m, 2H, Ha-6, Ha-7); 1.14 (s, 3H, H-15); 1.33–1.43 (m, 1H, Ha-9); 1.63 (dd, *J*(4a, 4e) = 13.4 Hz, *J*(4a, 3a) = 11.6 Hz, 1H, Ha-4); 1.70–1.89 (m, 4H, He-7, He-8, He-4, He-10); 2.01 (s, 3H, H-18); 3.50 (ddd, *J*(1a, 10a) = 11.2 Hz, *J*(1a, 6a) = 9.7 Hz, *J*(1a, 10e) = 4.2 Hz, 1H, Ha-1); 4.85 (ddd, *J*(3a, 4a) = 11.6 Hz, *J*(3a, 4e) = 2.4 Hz, *J*(3a, 14) = 1.0 Hz, 1H, Ha-3); 6.37 (d, *J*(14, 13) = 3.8 Hz, 1H, H-14); 6.59 (d, *J*(13, 14) = 3.8 Hz, 1H, H-13). ^13^C-NMR (CDCl_3_) (ppm) δ: 75.77 (d, C-1); 70.71 (d, C-3); 47.34 (t, C-4); 68.95 (s, C-5); 49.31 (d, C-6); 22.40 (t, C-7); 34.32 (t, C-8); 31.17 (d, C-9); 40.97 (t, C-10); 136.94 (s, C-11); 138.64 (s, C-12); 110.89 (d, C-13); 120.92 (d, C-14); 22.41 (q, C-15); 20.81 (q, C-16); 167.39 (s, C-17); 22.03 (q, C-18). HR MS: 323.1547 (M^+^, C_17_H_25_NO_3_S^+^; calc. 323.1550). ${\left[\alpha \right]}_{D}^{27.8}$ = +20.5 (c 0.16, EtOH).

*2,2,2-Trifluoro-N-(5-((2R,4R,4aR,7R,8aR)-4-hydroxy-4,7-dimethyloctahydro-2H-chromen-2-yl)thiophen-2-yl)acetamide*, (**4*R***)-**11**. The reaction of amine (**4*R***)-**9** (0.110 g) and trifluoroacetic anhydride (0.082 g) in the presence of 4-DMAP (0.001 g) for 2 h resulted in the yield of (**4*R***)-**11** (0.043 g, 30%). ^1^H-NMR (CDCl_3_) (ppm) δ: 0.80-0.88 (m, 1H, Ha-8); 0.82 (d, *J*(16, 9a) = 6.5 Hz, 3H, H-16); 0.94 (ddd, *J*(10a, 10e) = *J*(10a, 9a) = 12.1 Hz, *J*(10a, 1a) = 11.2 Hz, 1H, Ha-10); 1.03–1.12 (m, 2H, Ha-6, Ha-7); 1.14 (s, 3H, H-15); 1.33–1.43 (m, 1H, Ha-9); 1.63 (dd, *J*(4a, 4e) = 13.4 Hz, *J*(4a, 3a) = 11.6 Hz, 1H, Ha-4); 1.70–1.89 (m, 4H, He-7, He-8, He-4, He-10); 3.16 (ddd, *J*(1a, 10a) = 11.2 Hz, *J*(1a, 6a) = 9.7 Hz, *J*(1a, 10e) = 4.2 Hz, 1H, Ha-1); 4.50 (ddd, *J*(3a, 4a) = 11.6 Hz, *J*(3a, 4e) = 2.4 Hz, *J*(3a, 14) = 1.0 Hz, 1H, Ha-3); 6.64 (d, *J*(14, 13) = 3.7 Hz, 1H, H-14); 6.67 (d, *J*(13, 14) = 3.7 Hz, 1H, H-13). ^13^C-NMR (CDCl_3_) (ppm) δ: 77.50 (d, C-1); 72.35 (d, C-3); 49.34 (t, C-4); 69.34 (s, C-5); 51.31 (d, C-6); 22.40 (t, C-7); 34.32 (t, C-8); 31.19 (d, C-9); 41.03 (t, C-10); 135.70 (s, C-11); 139.15 (s, C-12); 114.76 (d, C-13); 121.00 (d, C-14); 22.81 (q, C-15); 20.81 (q, C-16); 153.39 (s, C-17); 115.21 (q, C-18). HR MS: 377.1265 (M^+^, C_17_H_22_NO_3_F_3_S^+^; calc. 377.1267). ${\left[\alpha \right]}_{D}^{29.1}$ = −27.1 (c 0.48, EtOH).

*2,2,2-Trifluoro-N-(5-((2R,4S,4aR,7R,8aR)-4-hydroxy-4,7-dimethyloctahydro-2H-chromen-2-yl)thiophen-2-yl)acetamide*, (**4*S***)-**11**. The reaction of amine (**4*S***)-**9** (0.052 g) and trifluoroacetic anhydride (0.039 g) in the presence of 4-DMAP (0.001 g) for 2 h resulted in the yield of (**4*S***)-**11** (0.055 g, 80%). ^1^H-NMR (CDCl_3_) (ppm) δ: 0.80-0.88 (m, 1H, Ha-8); 0.82 (d, *J*(16, 9a) = 6.5 Hz, 3H, H-16); 0.94 (ddd, *J*(10a, 10e) = *J*(10a, 9a) = 12.1 Hz, *J*(10a, 1a) = 11.2 Hz, 1H, Ha-10); 1.03–1.12 (m, 2H, Ha-6, Ha-7); 1.14 (s, 3H, H-15); 1.33–1.43 (m, 1H, Ha-9); 1.63 (dd, *J*(4a, 4e) = 13.4 Hz, *J*(4a, 3a) = 11.6 Hz, 1H, Ha-4); 1.70–1.89 (m, 4H, He-7, He-8, He-4, He-10); 3.55 (ddd, *J*(1a, 10a) = 11.2 Hz, *J*(1a, 6a) = 9.7 Hz, *J*(1a, 10e) = 4.2 Hz, 1H, Ha-1); 4.95 (ddd, *J*(3a, 4a) = 11.6 Hz, *J*(3a, 4e) = 2.4 Hz, *J*(3a, 14) = 1.0 Hz, 1H, Ha-3); 6.69 (d, *J*(14, 13) = 3.7 Hz, 1H, H-14); 6.73 (d, *J*(13, 14) = 3.7 Hz, 1H, H-13). ^13^C-NMR (CDCl_3_) (ppm) δ: 75.80 (d, C-1); 70.71 (d, C-3); 47.54 (t, C-4); 69.34 (s, C-5); 49.31 (d, C-6); 22.40 (t, C-7); 34.32 (t, C-8); 31.19 (d, C-9); 41.03 (t, C-10); 134.84 (s, C-11); 140.70 (s, C-12); 115.76 (d, C-13); 121.00 (d, C-14); 22.41 (q, C-15); 20.81 (q, C-16); 153.39 (s, C-17); 115.32 (q, C-18). HR MS: 377.1265 (M^+^, C_17_H_22_NO_3_F_3_S^+^; calc. 377.1267).

*General Procedure for the Preparation of Compounds***12–13.** To a solution of amine **9** (1.0 mmol) in EtOAc, acid chloride (1.0 mmol) in the presence of NEt_3_ (1 mmol) was added dropwise and the mixture was stirred at r.t. for 2 h. After the completion of the reaction, 10 mL of a saturated aq. NaHCO_3_ solution was added to the reaction mixture and extracted with ethyl acetate (3 × 10 mL). The organic layer was washed with brine (2 × 10 mL) and dried over anhydrous sodium sulphate. The organic layer was concentrated in a rotary evaporator and separated using an SiO_2_ column.

*N-(5-((2R,4R,4aR,7R,8aR)-4-Hydroxy-4,7-dimethyloctahydro-2H-chromen-2-yl)thiophen-2-yl)benzamide*, (**4*R***)-**12**. The reaction of amine (**4*R***)-**9** (0.130 g) and benzoyl chloride (0.064 g) in the presence of NEt_3_ (0.046 g) for 2 h resulted in the yield of (**4*R***)-**12** (0.056 g, 30%). ^1^H-NMR (CDCl_3_) (ppm) δ: 0.88–0.97 (m, 1H, Ha-8); 0.92 (d, *J*(16, 9a) = 6.5 Hz, 3H, H-16); 1.05 (ddd, *J*(10a, 10e) = *J*(10a, 9a) = 12.1 Hz, *J*(10a, 1a) = 11.2 Hz, 1H, Ha-10); 1.09–1.20 (m, 2H, Ha-6, Ha-7); 1.24 (s, 3H, H-15); 1.41–1.52 (m, 1H, Ha-9); 1.67 (dd, *J*(4a, 4e) = 13.4 Hz, *J*(4a, 3a) = 11.6 Hz, 1H, Ha-4); 1.72 (dm, *J*(8e, 8a) = 13.1 Hz, other *J* < 3.5 Hz, 1H, He-8); 1.77–1.83 (m, 1H, He-7); 1.95 (dd, *J*(4e, 4a) = 13.4 Hz, *J*(4e, 3a) = 2.4 Hz, 1H, He-4); 1.97 (dm, *J*(10e, 10a) = 12.1 Hz, other *J* < 4.5 Hz, 1H, He-10); 3.26 (ddd, *J*(1a, 10a) = 11.2 Hz, *J*(1a, 6a) = 9.7 Hz, *J*(1a, 10e) = 4.2 Hz, 1H, Ha-1); 4.62 (ddd, *J*(3a, 4a) = 11.6 Hz, *J*(3a, 4e) = 2.4 Hz, *J*(3a, 14) = 1.0 Hz, 1H, Ha-3); 6.60 (dd, *J*(14, 13) = 4.2 Hz, *J*(14, 3a) = 1.0 Hz, 1H, H-14); 6.73 (d, *J*(13, 14) = 4.2 Hz, 1H, H-13); 7.42–7.46 (m, 2H, H-20, H-22); 7.49–7.54 (m, 1H, H-21); 7.82–7.86 (m, 2H, H-19, H-23). ^13^C-NMR (CDCl_3_) (ppm) δ: 77.79 (d, C-1); 72.70 (d, C-3); 49.53 (t, C-4); 70.81 (s, C-5); 41.93 (d, C-6); 22.96 (t, C-7); 34.34 (t, C-8); 31.38 (d, C-9); 41.38 (t, C-10); 133.21 (s, C-11); 138.49 (s, C-12); 111.64 (d, C-13); 120.62 (d, C-14); 30.83 (q, C-15); 22.03 (q, C-16); 163.58 (s, C-17); 137.84 (s, C-18); 127.01 (d, C-19, C-23); 132.10 (d, C-21); 128.86 (d, C-20, C-22). HR MS: 385.1710 (M^+^, C_22_H_27_O_3_NS^+^; calc. 385.1706). ${\left[\alpha \right]}_{D}^{28.1}$ = +22.38 (c 0.143, EtOH).

*N-(5-((2R,4S,4aR,7R,8aR)-4-Hydroxy-4,7-dimethyloctahydro-2H-chromen-2-yl)thiophen-2-yl)benzamide*, (**4*S***)-**12**. The reaction of amine (**4*S***)-**9** (0.040 g) and benzoyl chloride (0.021 g) in the presence of NEt_3_ (0.015 g) for 2 h resulted in the yield of (**4*S***)-**12** (0.020 g, 40%). ^1^H-NMR (CDCl_3_) (ppm) δ: 0.88–0.97 (m, 1H, Ha-8); 0.92 (d, *J*(16, 9a) = 6.5 Hz, 3H, H-16); 1.05 (ddd, *J*(10a, 10e) = *J*(10a, 9a) = 12.1 Hz, *J*(10a, 1a) = 11.2 Hz, 1H, Ha-10); 1.09–1.20 (m, 2H, Ha-6, Ha-7); 1.24 (s, 3H, H-15); 1.41–1.52 (m, 1H, Ha-9); 1.67 (dd, *J*(4a, 4e) = 13.4 Hz, *J*(4a, 3a) = 11.6 Hz, 1H, Ha-4); 1.72 (dm, *J*(8e, 8a) = 13.1 Hz, other *J* < 3.5 Hz, 1H, He-8); 1.77–1.83 (m, 1H, He-7); 1.95 (dd, *J*(4e, 4a) = 13.4 Hz, *J*(4e, 3a) = 2.4 Hz, 1H, He-4); 1.97 (dm, *J*(10e, 10a) = 12.1 Hz, other *J* < 4.5 Hz, 1H, He-10); 3.57 (ddd, *J*(1a, 10a) = 11.2 Hz, *J*(1a, 6a) = 9.7 Hz, *J*(1a,10e) = 4.2 Hz, 1H, Ha-1); 4.96 (ddd, *J*(3a, 4a) = 11.6 Hz, *J*(3a, 4e) = 2.4 Hz, *J*(3a, 14) = 1.0 Hz, 1H, Ha-3); 6.60 (dd, *J*(14, 13) = 4.2 Hz, *J*(14, 3a) = 1.0 Hz, 1H, H-14); 6.73 (d, *J*(13, 14) = 4.2 Hz, 1H, H-13); 7.42–7.46 (m, 2H, H-20, H-22); 7.49–7.54 (m, 1H, H-21); 7.82–7.86 (m, 2H, H-19, H-23). ^13^C-NMR (CDCl_3_) (ppm) δ: 75.72 (d, C-1); 70.75 (d, C-3); 47.60 (t, C-4); 69.39 (s, C-5); 49.28 (d, C-6); 22.44 (t, C-7); 34.29 (t, C-8); 31.25 (d, C-9); 41.19 (t, C-10); 133.99 (s, C-11); 138.31 (s, C-12); 111.70 (d, C-13); 120.60 (d, C-14); 28.12 (q, C-15); 22.11 (q, C-16); 163.57 (s, C-17); 138.40 (s, C-18); 127.03 (d, C-19, C-23); 131.96 (d, C-21); 128.70 (d, C-20, C-22). HR MS: 385.1710 (M^+^, C_22_H_27_O_3_NS^+^; calc. 385.1706). ${\left[\alpha \right]}_{D}^{29.1}$ = +4.54 (c 0.22, EtOH).

*N-(5-((2R,4R,4aR,7R,8aS)-4-Hydroxy-4,7-dimethyloctahydro-2H-chromen-2-yl)thiophen-2-yl)adamantane-1-carboxamide*, (**4*R***)-**13**. The reaction of amine (**4*R***)-**9** (0.055 g) and 1-adamantanecarbonyl chloride (0.040 g) in the presence of NEt_3_ (0.030 g) for 2 h resulted in the yield of (**4*R***)-**13** (0.016 g, 20%). ^1^H-NMR (CDCl_3_) (ppm) δ: 0.87–0.96 (m, 1H, Ha-8); 0.89 (d, *J*(16, 9a) = 6.5 Hz, 3H, H-16); 0.98–1.09 (m, 1H, Ha-10); 1.09–1.16 (m, 2H, Ha-6, Ha-7); 1.21 (s, 3H, H-15); 1.40–1.49 (m, 1H, Ha-9); 1.67–1.77 (m, 7H, Ha-4, 2H-21, 2H-23, 2H-27); 1.84–1.97 (m, 2H, He-8, He-7); 1.91 (bs, 6H, 2H-19, 2H-25, 2H-26); 1.98–2.01 (m, 2H, He-4, He-10); 2.06 (bs, 3H, H-20, H-22, H-24); 3.23 (ddd, *J*(1a, 10a) = 11.2 Hz, *J*(1a, 6a) = 9.7 Hz, *J*(1a, 10e) = 4.2 Hz, 1H, Ha-1); 4.57 (d, *J*(3a, 4a) = 11.6 Hz, 1H, Ha-3); 6.45 (d, *J*(14, 13) = 4.2 Hz, 1H, H-14); 6.68 (d, *J*(13, 14) = 4.2 Hz, 1H, H-13). ^13^C-NMR (CDCl_3_) (ppm) δ: 77.37 (d, C-1); 72.48 (d, C-3); 49.50 (t, C-4); 70.71 (s, C-5); 51.89 (d, C-6); 22.91 (t, C-7); 34.23 (t, C-8); 31.34 (d, C-9); 41.31 (t, C-10); 136.97 (s, C-11); 138.56 (s, C-12); 110.26 (d, C-13); 120.19 (d, C-14); 21.08 (q, C-15); 22.05 (q, C-16); 174.08 (s, C-17); 40.69 (s, C-18); 38.99(t, C-19, C-25, C-26); 27.89 (d, C-20, C-22, C-24); 36.23 (t, C-21, C-23, C-27). HR MS: 443.2481 (M^+^, C_26_H_37_O_3_NS^+^; calc. 443.2489). ${\left[\alpha \right]}_{D}^{27.1}$ = +7.9 (c 0.304, EtOH).

*N-(5-((2R,4S,4aR,7R,8aS)-4-Hydroxy-4,7-dimethyloctahydro-2H-chromen-2-yl)thiophen-2-yl)adamantane-1-carboxamide*, (**4*S***)-**13**. The reaction of amine (**4*S***)-**9** (0.290 g) and 1-adamantanecarbonyl chloride (0.297 g) in the presence of NEt_3_ (0.300 g) for 2 h resulted in the yield of (**4*S***)-**13** (0.065 g, 14%). ^1^H-NMR (CDCl_3_) (ppm) δ: 0.87–0.96 (m, 1H, Ha-8); 0.89 (d, *J*(16, 9a) = 6.5 Hz, 3H, H-16); 1.00 (dd, *J*(10a, 10e) = *J*(10a, 9a) = 12.1 Hz, 1H, Ha-10); 1.09–1.16 (m, 2H, Ha-6, Ha-7); 1.21 (s, 3H, H-15); 1.40–1.49 (m, 1H, Ha-9); 1.66–1.75 (m, 7H, Ha-4, 2H-21, 2H-23, 2H-27); 1.77–1.89 (m, 2H, He-8, He-7); 1.90 (bs, 6H, 2H-19, 2H-25, 2H-26); 1.92–1.95 (m, 2H, He-4, He-10); 2.05 (bs, 3H, H-20, H-22, H-24); 3.53 (ddd, *J*(1a, 10a) = 11.2 Hz, *J*(1a, 6a) = 9.7 Hz, *J*(1a, 10e) = 4.2 Hz, 1H, Ha-1); 4.91 (dd, *J*(3a, 4a) = 11.6 Hz, *J*(3a, 4e) = 2.4 Hz, 1H, Ha-3); 6.44 (d, *J*(14, 13) = 4.2 Hz, 1H, H-14); 6.67 (d, *J*(13, 14) = 4.2 Hz, 1H, H-13). ^13^C-NMR (CDCl_3_) (ppm) δ: 75.51 (d, C-1); 70.68 (d, C-3); 47.63 (t, C-4); 69.43 (s, C-5); 49.18 (d, C-6); 22.47 (t, C-7); 34.33 (t, C-8); 31.15 (d, C-9); 41.08 (t, C-10); 137.70 (s, C-11); 138.37 (s, C-12); 110.21 (d, C-13); 120.24 (d, C-14); 28.11 (q, C-15); 22.10 (q, C-16); 174.06 (s, C-17); 40.63 (s, C-18); 39.11(t, C-19, C-25, C-26); 27.86 (d, C-20, C-22, C-24); 36.25 (t, C-21, C-23, C-27). HR MS: 443.2481 (M^+^, C_26_H_37_O_3_NS^+^; calc. 443.2489).${\left[\alpha \right]}_{D}^{25.0}$ = +19.3 (c 0.114, EtOH).

### 3.2. Tdp1 Assay

The pET 16B-Tdp1 was kindly provided by Dr. K.W. Caldecott (University of Sussex, United Kingdom). Recombinant Tdp1 was expressed in *Escherichia coli* BL21(DE3) and was purified by chromatography on Ni-chelating resin and phosphocellulose P11, as described in [29].

Real-time Tdp1 activity measurements were carried out as described in [9]. In brief, Tdp1-biosensor (50 nM) was incubated in a buffer that contains 50 mM Tris-HCl pH 8.0, 50 mM NaCl, and 7 mM β-mercaptoethanol (200 μL). The mixture was then supplemented with purified Tdp1 (1.3 nM) and various concentrations of the inhibitor. Fluorescence measurements (Ex_485_/Em_520_ nm) were carried out during the linear phase of reaction (from 0 to 8 min) every 55 s. The reactions were incubated at a constant temperature of 26 °C using a POLARstar OPTIMA fluorimeter (BMG LABTECH). The influence of the compounds was evaluated by comparing the fluorescence increase rate in their presence to that of 1.5% DMSO control wells. The data were imported into the MARS Data Analysis 2.0 program (BMG LABTECH), and IC_50_ values were calculated by non-linear curve fitting. Tdp1-biosensor 5′-(5,6 FAM-aac gtc agg gtc ttc c-BHQ1)-3′ was synthesised in the Laboratory of Biomedical Chemistry, Institute of Chemical Biology and Fundamental Medicine, Novosibirsk, Russia.

### 3.3. Binding Studies

Synthetic DNA encoding human Tdp1 (residues 149-608 [2]) was cloned into pET-28a(+) (GenScript), which was then transformed into *Escherichia coli* BL21(DE3) for recombinant protein production. Protein production was induced with 1 mM IPTG at 28 °C with overnight incubation. Tdp1 was purified using affinity and size exclusion chromatography. Fluorescence was measured using a PerkinElmer EnSpire Multimode Reader. Tdp1 concentration was 10 μM and compound concentrations were 1 μM, 2.5 μM, 5 μM, 10 μM, 15 μM, 20 μM, 30 μM, 50 μM, 100 μM, 200 μM, 350 μM, 500 μM, and 750 μM. Buffer was 20 mM Tris and 250 mM NaCl (pH 8). Excitation wavelength was 280 nm and intrinsic fluorescence was measured between 300 and 450 nm. Control experiments with Tdp1 or compound on its own were also conducted. Background fluorescence arising from the compounds was subtracted from the final spectrum. The total volume per well was 30 μL. Dissociation constant (*K*_D_) values were calculated using the following formula (1), which takes non-specific binding into account [32].

$$I\;=\;\frac{I_{\max\;}\times \left[L_{T}\right]}{K_{D}+\left[L_{T}\right]\;}+N_{s}\left[L_{T}\right]$$

I indicates changes in fluorescence intensity from the titrations, I_max_ indicates the maximum fluorescence intensity change, [L_T_] is the titrated ligand concentration, and *N*_s_ is the non-specific term. Non-linear curve fitting was conducted using SigmaPlot 13.0 (Systat Software, San Jose, CA, USA). Experiments were conducted in triplicate and the errors shown are standard derivations.

### 3.4. Molecular Modelling Methods

Molecular modelling and the generation of molecular descriptors of Tdp1 inhibitors were conducted as described in [10]. Briefly, the crystal structure of Tdp1 was used for docking (PDB ID: 1MU7) [33] with the GOLD v5.4 software and the molecular descriptors were generated using QikProp v5.2 [34,35].

## 4. Conclusions

In this study, we reported a new class of Tdp1 inhibitors that are based on the (-)-isopulegol-derived octahydro-2*H*-chromene scaffold. By using a three-step reaction, a series of chiral heterocyclic compounds that contain the octahydro-2*H*-chromene scaffold and different amide substituents were synthesised. These structural series of compounds were found to be good inhibitors and binders of Tdp1, with IC_50_ and *K*_D_ values in the low micromolar concentration range. In particular, the octahydro-2*H*-chromen-4-ol derivative with 1-adamantane moiety (**4*S***)-**13** appeared to be the most potent inhibitor of Tdp1 among this series, with an IC_50_ value of 1.24 µM. The plausible binding modes of these compounds to Tdp1 were also predicted, which found that these compounds may block the access of substrates to the enzyme active site. Overall, our results helped establish a structural-activity relationship for these novel octahydro-2*H*-chromene-based Tdp1 inhibitors. This information will be useful for future inhibitor development work for Tdp1.

## Figures and Tables

**Table 1:** Inhibitory Potency (IC_50_) and Binding Affinity (*K*_D_) of Compounds **8–13** Against Tdp1.

Compound			IC_50_, µM		*K*_D_, μM	
		**R**	(**4*S***)	(**4*R***)	(**4*S***)	(**4*R***)
**8**	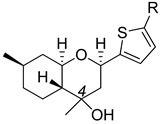	NO_2_-	>15	>15	30.6 ± 9.3	23.3 ± 7.4
**9**		NH_2_-	>15	>15	n.d.	n.d.
**10**	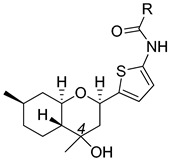	CH_3_-	5.8 ± 3.0	2.9 ± 0.8	n.d.	n.d.
**11**		CF_3_-	1.4 ± 0.3	4.0 ± 0.4	2.0 ± 1.2	19.8 ± 2.4
**12**		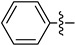	5.0 ± 1.5	3.3 ± 0.2	24.5 ± 5.7	n.d.
**13**		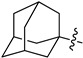	1.24 ± 0.02	2.8 ± 0.6	17.9 ± 3.4	12.4 ± 7.5
Fur *			1.2 ± 0.3		n.d.	

* Furamidine was used as a positive control. n.d. = not determined.

**Table 2:** Results of the Scoring Functions (GS, CS, ChemPLP, and ASP) for Compounds **8–17** with Tdp1. The IC_50_ Values of Compounds **8–13** with Tdp1 were Also Included.

Compound	GS	CS	ChemPLP	ASP	IC_50_ (μM)
(**4*R***)-**8**	40.1	26.0	47.9	27.4	>15
(**4*S***)-**8**	41.0	25.3	45.0	26.6	>15
(**4*R***)-**9**	41.5	25.0	41.1	27.2	>15
(**4*S***)-**9**	42.2	25.5	45.5	29.0	>15
(**4*R***)-**10**	42.3	25.9	50.7	28.9	2.9 ± 0.8
(**4*S***)-**10**	44.0	25.2	49.4	28.4	5.8 ± 3.0
(**4*R***)-**11**	39.6	23.8	51.8	34.4	4.0 ± 0.4
(**4*S***)-**11**	44.0	25.2	50.6	31.3	1.4 ± 0.3
(**4*R***)-**12**	55.8	30.3	57.2	29.1	3.3 ± 0.2
(**4*S***)-**12**	56.0	30.8	56.4	29.8	5.0 ± 1.5
(**4*R***)-**13**	51.5	32.3	57.1	30.5	2.8 ± 0.6
(**4*S***)-**13**	52.5	31.4	56.1	30.4	1.24 ± 0.02
(**4*R***)-**14**	60.9	30.9	60.4	31.3	-
(**4*S***)-**14**	62.5	29.6	61.3	32.8	-
(**4*R***)-**15**	50.2	30.5	64.8	32.6	-
(**4*S***)-**15**	53.6	28.2	59.6	28.5	-
(**4*R***)-**16**	48.7	23.2	51.6	25.9	-
(**4*S***)-**16**	48.8	21.5	48.5	27.5	-
(**4*R***)-**17**	49.2	21.9	49.3	25.1	-
(**4*S***)-**17**	51.2	22.2	47.9	27.7	-

## Supplementary Materials

The Supplementary Materials are available online.
