# Supplementary material for: A Novel Class of Tyrosyl-DNA Phosphodiesterase 1 Inhibitors That Contains the Octahydro-2H-chromen-4-ol Scaffold
Source: Molecules. 2018 Sep 26;23(10):2468. doi: 10.3390/molecules23102468 (PMC6222798; doi:10.3390/molecules23102468)
Supplement: Supplementary file 1 [file molecules-23-02468-s001.pdf]

Supplementary information

A Novel Class of (-)-Isopulegol Based Tyrosyl-DNA Phosphodiesterase 1 Inhibitors

Nikolai S. Li-Zhulanov[1,2], Alexandra L. Zakharenko[3], Arina A. Chepanova[3], Jinal Patel[4], Ayesha Zafar[4], Konstantin P. Volcho[1,2,*], Nariman F. Salakhutdinov[1,2], Jóhannes Reynisson[4,*], Ivanhoe K.H. Leung[4,*], and Olga I. Lavrik[2,3]

1. N. N. Vorozhtsov Novosibirsk Institute of Organic Chemistry, Siberian Branch of the Russian Academy of Sciences, 9, Akademika Lavrentieva Ave., Novosibirsk, 630090, Russian Federation
2. Novosibirsk State University, Pirogova 2, Novosibirsk, 630090, Russian Federation
3. Novosibirsk Institute of Chemical Biology and Fundamental Medicine, Siberian Branch of the Russian Academy of Sciences, 8, Akademika Lavrentieva Ave., Novosibirsk, 630090, Russian Federation
4. School of Chemical Sciences, The University of Auckland, Private Bag 92019, Victoria Street West, Auckland 1142, New Zealand

Table S1. Calculated molecular descriptors for the selected compounds.

**Compound**	MW(g/mol)	HB Donor	HB Acceptor	Log P	PSA($Å^2$)	Rotatable Bonds
**(4*R*)-8**	311.4	1	3.4	3.1	72.9	2
**(4*S*)-8**	311.4	1	3.4	3.1	71.8	2
**(4*R*)-9**	281.4	2.5	3.4	2.8	53.2	1
**(4*S*)-9**	281.4	2.5	3.4	2.8	51.4	1
**(4*R*)-10**	385.5	2	4.9	4.2	65.2	3
**(4*S*)-10**	385.5	2	4.9	4.3	63.1	3
**(4*R*)-11**	323.5	2	4.9	3.0	68.9	2
**(4*S*)-11**	323.5	2	4.9	3.3	64.6	2
**(4*R*)-12**	377.4	2	4.9	3.9	64.9	2
**(4*S*)-12**	377.4	2	4.9	4.1	62.9	2
**(4*R*)-13**	443.6	2	4.9	5.1	62.8	3
**(4*S*)-13**	443.6	2	4.9	5.1	60.8	3
**(4*R*)-14**	435.6	2	4.9	5.5	61.2	3
**(4*S*)-14**	435.6	2	4.9	5.3	62.3	3
**(4*R*)-15**	435.6	2	4.9	5.2	64.1	3
**(4*S*)-15**	435.6	2	4.9	5.3	64.4	3
**(4*R*)-16**	351.5	2	4.9	3.9	61.9	3
**(4*S*)-16**	351.5	2	4.9	3.8	63.4	3
**(4*R*)-17**	365.5	2	4.9	4.2	59.4	3
**(4*S*)-17**	365.5	2	4.9	4.1	60.9	3

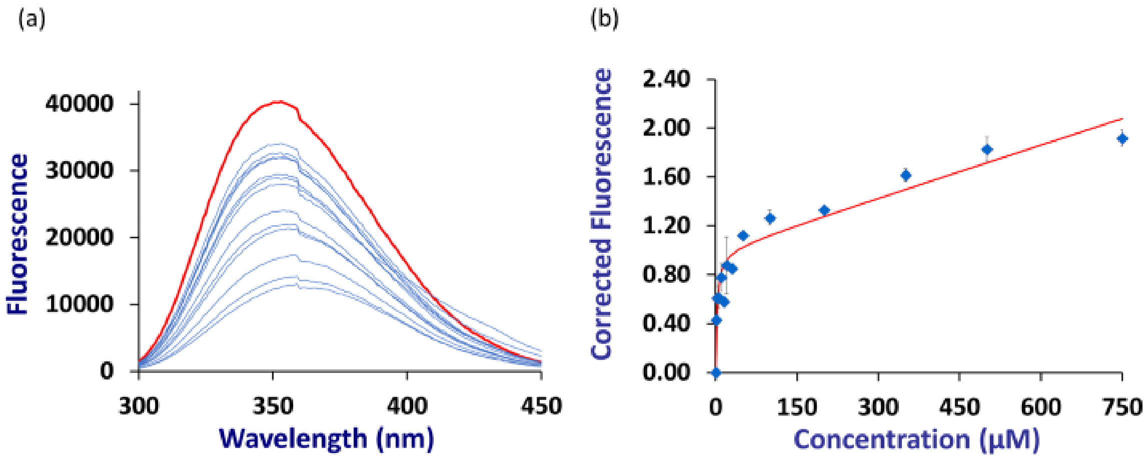

Supplementary Figure S1: (a) Changes in intrinsic fluorescence intensity of Tdp1 (10 µM) upon the addition of compound **8** (4*S*) (1 µM, 2.5 µM, 5 µM, 10 µM, 15 µM, 20 µM, 30 µM, 50 µM, 100 µM, 200 µM, 350 µM, 500 µM and 750 µM). Buffer was 20 mM Tris and 250 mM NaCl (pH 8). Excitation wavelength was 280 nm and intrinsic fluorescence was measured between 300 and 450 nm; (b) Non-linear curve fitting of the fluorescence data. The K_D was 30.6 ± 9.33 µM. Experiments were conducted in triplicate.

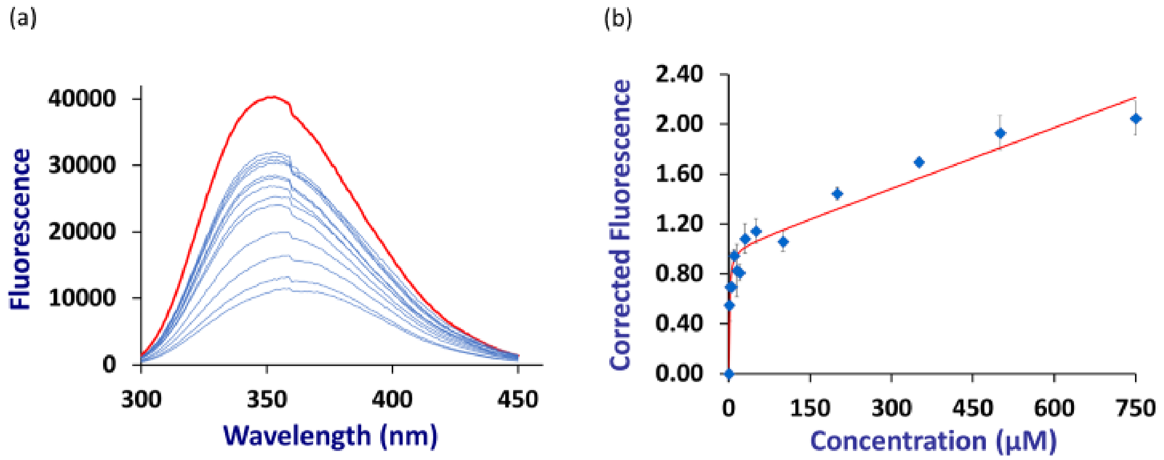

Supplementary Figure S2: (a) Changes in intrinsic fluorescence intensity of Tdp1 (10 µM) upon the addition of compound 8 (4*R*) (1 µM, 2.5 µM, 5 µM, 10 µM, 15 µM, 20 µM, 30 µM, 50 µM, 100 µM, 200 µM, 350 µM, 500 µM and 750 µM). Buffer was 20 mM Tris and 250 mM NaCl (pH 8). Excitation wavelength was 280 nm and intrinsic fluorescence was measured between 300 and 450 nm; (b) Non-linear curve fitting of the fluorescence data. The K_D was 23.3 ± 7.4 µM. Experiments were conducted in triplicate.

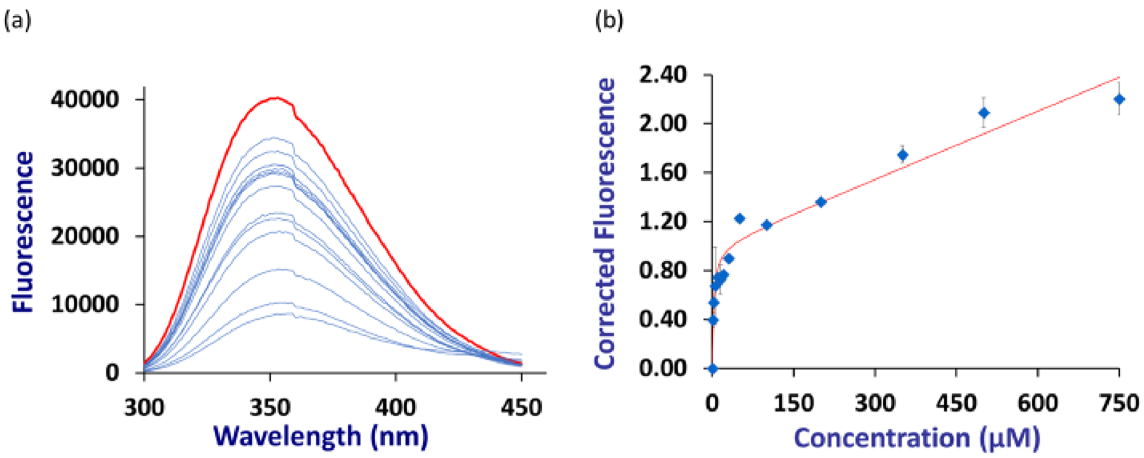

Supplementary Figure S3: (a) Changes in intrinsic fluorescence intensity of Tdp1 (10 µM) upon the addition of compound 11 (4S) (1 µM, 2.5 µM, 5 µM, 10 µM, 15 µM, 20 µM, 30 µM, 50 µM, 100 µM, 200 µM, 350 µM, 500 µM and 750 µM). Buffer was 20 mM Tris and 250 mM NaCl (pH 8). Excitation wavelength was 280 nm and intrinsic fluorescence was measured between 300 and 450 nm; (b) Non-linear curve fitting of the fluorescence data. The K_D was 2.0 ± 1.2 µM. Experiments were conducted in triplicate.

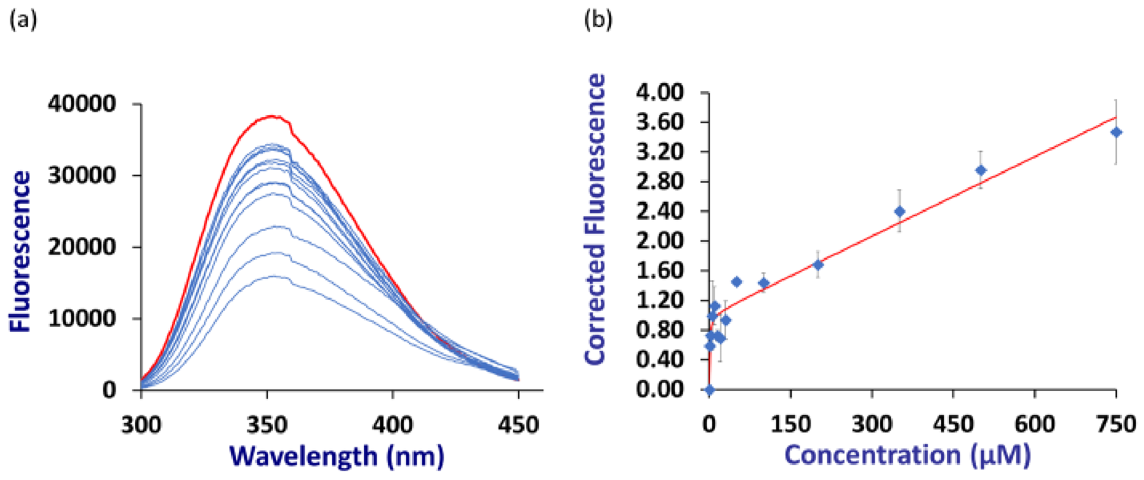

Supplementary Figure S4: (a) Changes in intrinsic fluorescence intensity of Tdp1 (10 µM) upon the addition of compound 11 (4R) (1 µM, 2.5 µM, 5 µM, 10 µM, 15 µM, 20 µM, 30 µM, 50 µM, 100 µM, 200 µM, 350 µM, 500 µM and 750 µM). Buffer was 20 mM Tris and 250 mM NaCl (pH 8). Excitation wavelength was 280 nm and intrinsic fluorescence was measured between 300 and 450 nm; (b) Non-linear curve fitting of the fluorescence data. The K_D was 19.8 ± 2.4 µM. Experiments were conducted in triplicate.

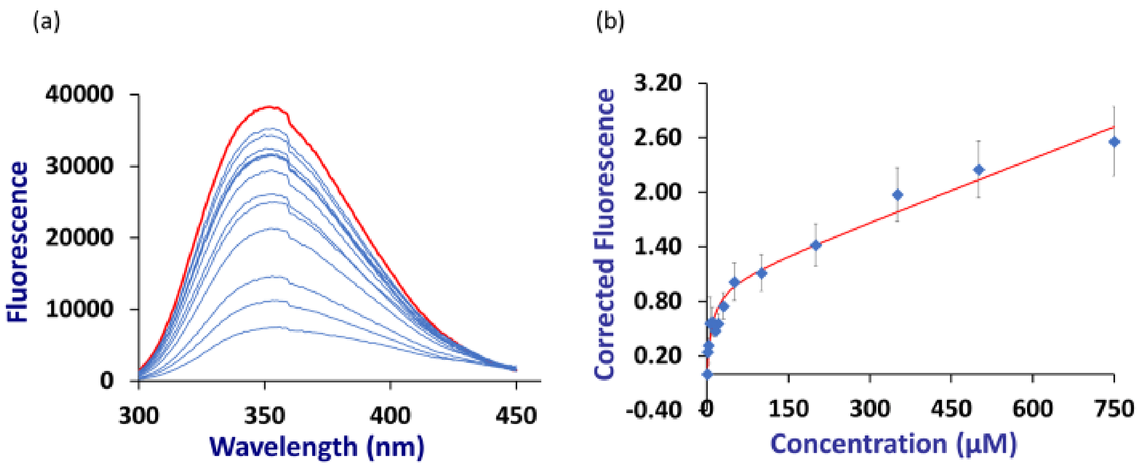

Supplementary Figure S5: (a) Changes in intrinsic fluorescence intensity of Tdp1 (10 µM) upon the addition of compound 12 (4S) (1 µM, 2.5 µM, 5 µM, 10 µM, 15 µM, 20 µM, 30 µM, 50 µM, 100 µM, 200 µM, 350 µM, 500 µM and 750 µM). Buffer was 20 mM Tris and 250 mM NaCl (pH 8). Excitation wavelength was 280 nm and intrinsic fluorescence was measured between 300 and 450 nm; (b) Non-linear curve fitting of the fluorescence data. The K_D was 24.5 ± 5.7 µM. Experiments were conducted in triplicate.

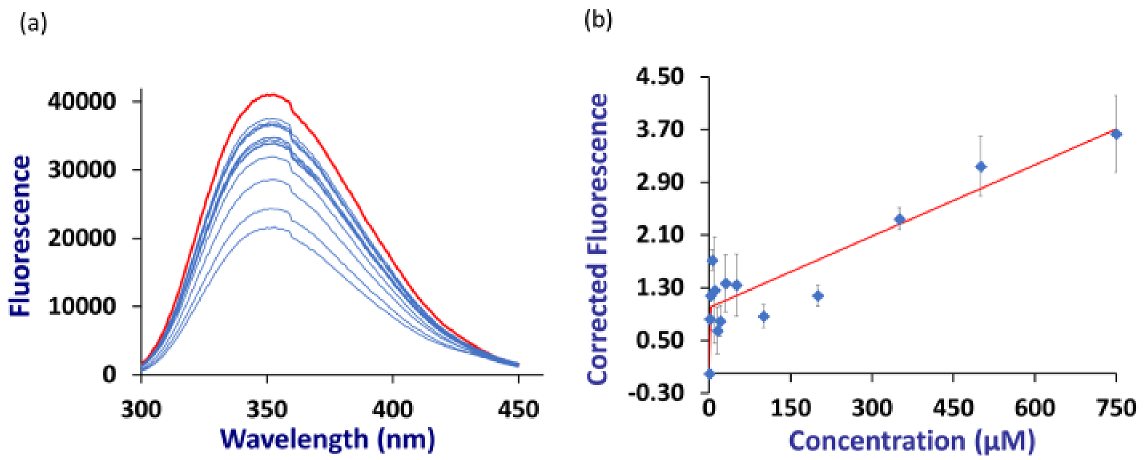

Supplementary Figure S6: (a) Changes in intrinsic fluorescence intensity of Tdp1 (10 µM) upon the addition of compound 13 (4S) (1 µM, 2.5 µM, 5 µM, 10 µM, 15 µM, 20 µM, 30 µM, 50 µM, 100 µM, 200 µM, 350 µM, 500 µM and 750 µM). Buffer was 20 mM Tris and 250 mM NaCl (pH 8). Excitation wavelength was 280 nm and intrinsic fluorescence was measured between 300 and 450 nm; (b) Non-linear curve fitting of the fluorescence data. The K_D was 17.9 ± 3.4 µM. Experiments were conducted in triplicate.

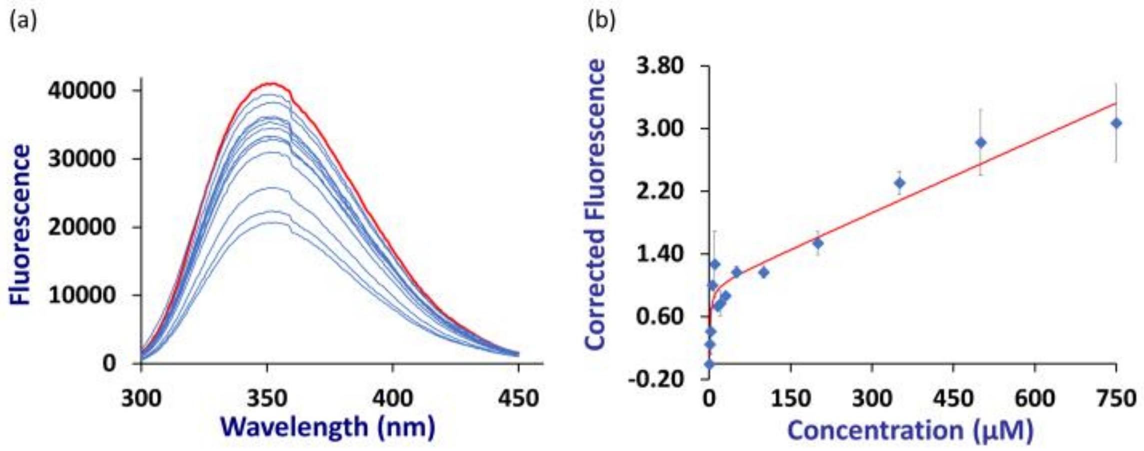

Supplementary Figure S7: (a) Changes in intrinsic fluorescence intensity of Tdp1 (10 µM) upon the addition of compound 13 (4R) (1 µM, 2.5 µM, 5 µM, 10 µM, 15 µM, 20 µM, 30 µM, 50 µM, 100 µM, 200 µM, 350 µM, 500 µM and 750 µM). Buffer was 20 mM Tris and 250 mM NaCl (pH 8). Excitation wavelength was 280 nm and intrinsic fluorescence was measured between 300 and 450 nm; (b) Non-linear curve fitting of the fluorescence data. The K_D was 12.4 ± 7.5 µM. Experiments were conducted in triplicate.
